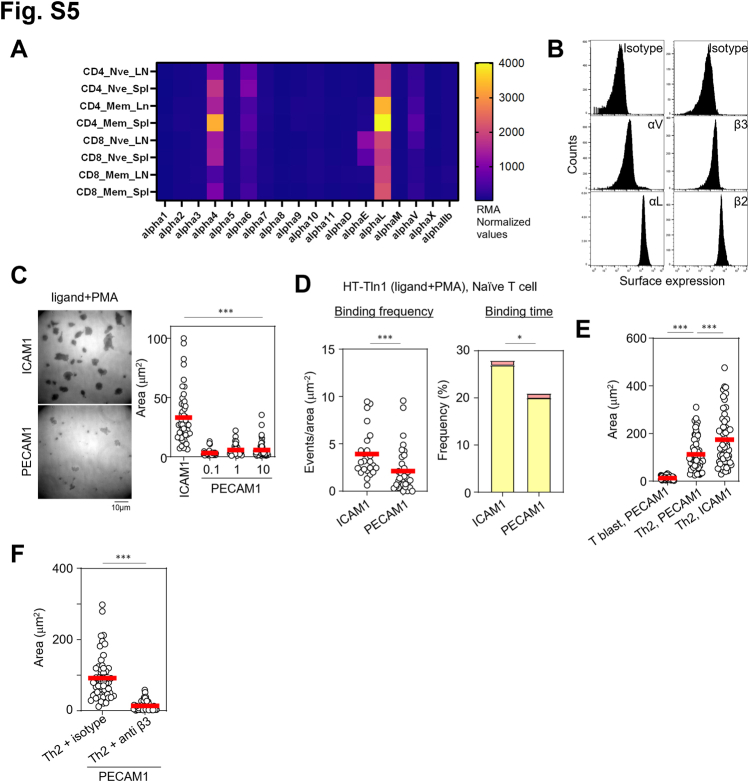# The nonconserved integrin cytoplasmic region determines integrin subtype–specific characteristics by modulating talin1 binding kinetics

**DOI:** 10.1016/j.jbc.2025.110793

**Published:** 2025-10-07

**Authors:** Naoyuki Kondo, Kenji Fukui, Yuji Kamioka, Yoshihiro Ueda, Yoshiki Ikeda, Taiju Matsushita, Ryo Yazaki, Tatsuo Kinashi

**Affiliations:** 1 Department of Molecular Genetics, Institute of Biomedical Science, Kansai Medical University, Osaka, Japan; 2 Department of Food Science and Nutrition, Nara Women’s University, Nara, Japan; 3 Graduate School of Pharmaceutical Sciences, Kyushu University, Fukuoka, Japan; 4 Institute for Advanced Study, Kyushu University, Fukuoka, Japan

**Keywords:** talin1, single-molecule imaging, LFA1, α4β7, αVβ3

## Abstract

Talin governs integrin adhesion by binding to the cytoplasmic tail of integrin β subunits, but the effects of integrin subtype–specific variations on talin interactions remain unclear. Here, we identify a nonconserved region within the cytoplasmic tail of integrin, termed the WN linker, that modulates talin1 binding kinetics and integrin adhesiveness. Single-molecule imaging in live lymphocytes revealed that talin1 bound more strongly to β2 than β7 integrin, with higher off-rates in β7 *in vivo*. This difference was due to the unique NND sequence in the β2 WN linker compared with KQDS in the β7 WN linker. Structural and biochemical analyses showed that NND established a tighter interaction with talin, whereas KQDS bent, narrowing the interaction area and weakening the interaction. Substituting the NND sequence in β2 with KQDS impaired inside–out signaling– and ligand binding–induced conformational activation of LFA1. Multiple sequence alignment and single-molecule binding analyses revealed that the NND sequence is highly conserved only in mammalian β2 integrins, and that the second asparagine in NND, a residue absent in nonmammalian β2 integrins and other integrins, plays a key role in talin1 binding. Parallel observations in β3 integrins reinforced the pivotal role of the WN linker in modulating integrin–talin affinity. These observations highlight the WN linker as a novel regulator of integrin–talin binding strength and adhesiveness diversity.

Integrins are heterodimeric transmembrane receptors composed of α- and β-subunits, which play pivotal roles in maintaining homeostasis and mediating progression of cancer and inflammatory diseases (1, 2). The functional diversity of integrins arises from their specificity for ligands, such as extracellular matrix proteins and cell-surface molecules on interacting cells, thus enabling their characteristic biological activities. Despite the diversity of ligand binding, most integrins commonly interact with talin, a key cytoskeletal adapter protein that activates integrins by inducing conformational changes to their active state (3). This integrin–talin interaction links integrins to the actin cytoskeleton, promoting stable cell adhesion (3).

Talin binds to the cytoplasmic tail (CT) of integrin β-subunits, including β1, β2, β3, β5, and β7 (4, 5). This interaction is mediated mainly by the conserved Trp residue (*e.g.*, W747 in human β2) and the proximal Asn-Pro-x-Tyr/Phe (NPxY/F) motif (*e.g.*, N751–P752–L753–F754 in human β2), which engages the F3 subdomain of the N-terminal four-point-one, ezrin, radixin, moesin (FERM) domain of talin (6, 7, 8, 9). The binding of talin to integrins is regulated by bidirectional signaling pathways: inside–out signaling, triggered by T-cell receptor or chemokine receptor activation, leading to Rap1-mediated integrin activation *via* talin1 recruitment to the plasma membrane (10, 11); outside–in signaling, initiated by ligand binding to integrins, also driving Rap1 activation to promote talin1 binding, leading to cytoskeletal reorganization (6, 12, 13, 14, 15). Our previous work demonstrated in living cells that talin1 directly interacts with W747 and/or the NPxY/F motif within the β2-CT to mediate LFA1 (αL/β2)–intercellular adhesion molecule 1 (ICAM1)–dependent adhesion (6). Moreover, full-length talin1, but not its FERM domain alone, is critical for robust integrin activation through its C-terminal ROD domain, which links to the actin cytoskeleton (6). These findings underscore the importance of force transduction through full-length talin1 for promoting the active integrin conformation necessary for stable adhesion. On the other hand, recent intramolecular FRET analyses by Li *et al.* (16) revealed that the binding to ligand, such as fibronectin, can trigger conformational changes in α5/β1 and α4/β1 integrins, and that the talin1 FERM domain does not strongly contribute to this activation, suggesting the existence of alternative ligand-dominant activation mechanisms; however, it remains unclear whether such mechanisms extend to other integrin subtypes.

Bidirectional signaling and traction force from talin on integrins are thought to be prerequisites for overcoming the large energy barrier for integrin conformational changes (17, 18), although experimental evidence for this hypothesis is limited. Recently, we found that slow rolling and arrest adhesion of T cells on ICAM1—a ligand for αL/β2 integrin LFA1—under shear flow, which is mediated by the active state of LFA1, requires both talin1 and Rap1 (15). Talin recruitment occurring concurrently with deceleration of rolling velocities suggests simultaneous engagement of ICAM1 and talin1 by LFA1 during slow rolling under shear flow. This finding highlights the importance of talin1 interaction and force transmission for the formation of the active conformations suitable for slow rolling and arrest adhesion.

Interestingly, the talin requirement for mucosal vascular addressin cell adhesion molecule 1 (MAdCAM1)–α4/β7 integrin–dependent adhesion under conditions of shear flow is distinct from that for ICAM1–LFA1—rolling and tethering, but not arrest adhesion to MAdCAM1 does not require talin1 and Rap1; transition from rolling to arrest adhesion is gradual, whereas that in LFA1 is steep (15). While these differences seem to be partly attributable to intrinsic structural properties, their ligand types and affinity to talin1, the molecular basis for differences in integrin-specific bidirectionality remains unclear.

Here, we investigated the functional divergence of talin interactions across integrins to elucidate the determinants of integrin type–specific adhesion. Using an *in vivo* single-molecule talin1 binding system (6), we found that talin1 binds more strongly to β2 integrin than to β3 and β7 integrins in both naïve and activated T cells. To eliminate the confounding effects of ligand specificity and integrin expression level, we engineered chimeric integrins, where β2-CT was replaced with that from β3 or β7. These chimeric integrins attenuated cell adhesiveness and reduced talin1 binding kinetics, recapitulating the β3 and β7 phenotypes, respectively. Furthermore, we identified the characteristic linker region between conserved Trp and NPxY/F motif, named WN linker, which controls talin1 binding kinetics and cell adhesiveness.

These findings provide critical insights into the molecular basis of integrin regulation, with implications for diverse integrin-mediated cell adhesion and signaling processes, which are applicable for development of integrin type–specific drugs or therapeutics.

## Results

### Differences in integrin-dependent adhesiveness

**Figure 1:**
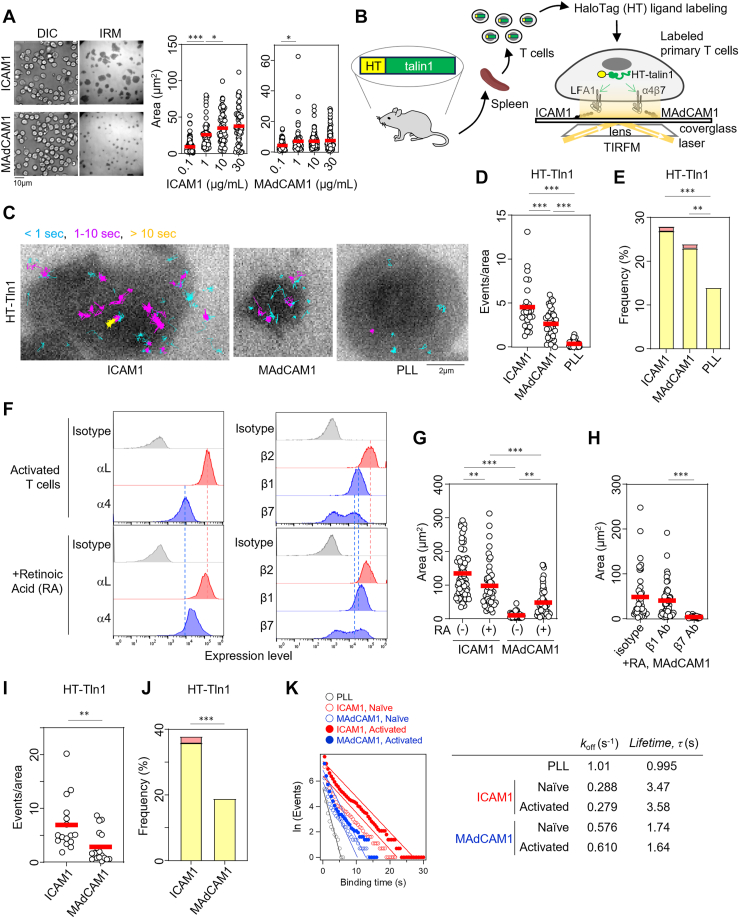
**Cell adhesion and talin1 binding kinetics of α4/β7 are weaker than those of LFA1 in primary T cells.***A*, ligand-dependent adhesion of T cells. ICAM1 and MAdCAM1 serve as ligands of LFA1 and α4/β7, respectively. PMA was used for the stimulation of inside–out signaling. Representative images at a ligand concentration of 10 μg/ml are shown. ICAM1 (0.1 μg/ml: *n* = 75; 1 μg/ml: *n* = 59; 10 μg/ml: *n* = 72; and 30 μg/ml: *n* = 58). MAdCAM1 (0.1 μg/ml: *n* = 63; 1 μg/ml: *n* = 84; 10 μg/ml: *n* = 119; and 30 μg/ml: *n* = 87). A representative of more than two independent experiments is shown. *B*, schematic representation of the experimental system used to measure talin1 binding kinetics to the CT *in vivo* using total internal reflection microscopy (TIRFM). Binding of HaloTag (HT)-fused talin1 (HT-talin1) to integrin-CT in an integrin–ligand binding–dependent manner was verified previously (6). *C*, single-molecule trajectories of HT-talin1 in primary T cells adhering to 10 μg/ml ICAM1-coated or 10 μg/ml MAdCAM1-coated dish with 100 ng/ml PMA. Poly-l-lysine (PLL)-coated dishes were used as integrin ligand-free controls. Talin1 trajectories are colored based on the time of appearance on TIRFM imaging. The *dark gray area* indicates the region of cell adhesion captured using IRM. *D*, binding frequency of talin1 to integrins (ICAM1: *n* = 27; MAdCAM1: *n* = 37; and PLL: *n* = 51). The number of events/trajectories was normalized to the single-cell area (events/area). *E*, binding time of talin1 (ICAM1: *n* = 1111; MAdCAM1: *n* = 506; and PLL: *n* = 224). Binding time, defined as the duration of single-molecule tracking, was divided into three categories: <1 s, 1 to 10 s, and >10 s. Frequencies of 1 to 10 s and >10 s categories are shown in *yellow* and *red*, respectively. *F*, expression of integrins on retinoic acid (RA)–supplemented activated T cells. T cells without RA supplementation are shown as a control. *G*, adhesiveness of activated T cells without (−) or with (+) RA supplementation to ICAM1 [(−): *n* = 65; (+): *n* = 45] or MAdCAM1 [(−): *n* = 46; (+): *n* = 54]. *H*, effects of blocking antibodies for β1 (*n* = 47) and β7 (*n* = 51) on T-cell adhesiveness in comparison with isotype control antibody (*n* = 44). *I*, binding frequency of talin1 in RA-supplemented activated T cells as in (*D*) (ICAM1: *n* = 16; MAdCAM1: *n* = 17). *J*, binding time of talin1 in RA-supplemented activated T cells as in (*E*) (ICAM1: *n* = 2780; MAdCAM1: *n* = 1627). *K*, dissociation rate constant of integrin–talin1 binding *in vivo*. The population with longer binding time was fitted linearly to calculate the rate constant, *k*_off_ (20). Statistical analyses were performed by one-way ANOVA with Tukey’s correction for multiple comparisons (*A*, *D*, *G*, and *H*), unpaired nonparametric two-sided Student’s *t* test (*I*), or Chi-squared test for trend (*E*, *J*). ∗*p* < 0.05, ∗∗*p* < 0.01, and ∗∗∗*p* < 0.001. CT, cytoplasmic tail; DIC, differential interference contrast; ICAM1, intercellular adhesion molecule 1; IRM, interference reflection microscopy; MAdCAM1, mucosal vascular addressin cell adhesion molecule 1; PMA, phorbol 12-myristate 13-acetate.

Previously, we described the differences between LFA1–ICAM1 interaction–dependent and α4/β7–MAdCAM1 interaction–dependent lymphocyte adhesion under shear flow conditions (15). To examine these differences under static conditions, we performed adhesion assays with ICAM1- or MAdCAM1-coated surfaces using primary T cells based on the area of adhesion (6). An increase in the surface ligand concentration led to a plateau of adhesiveness. However, even at maximum concentration, LFA1-dependent adhesion was stronger than α4/β7-dependent adhesion (Fig. 1*A*), suggesting that the LFA1–ICAM1 interaction is inherently stronger than the α4/β7–MAdCAM1 interaction.

### Single-molecule binding assay

To clarify the differences in talin1 binding properties between LFA1 and α4/β7 in primary T cells, we purified T cells from HaloTag-fused talin1 (HT-talin1) knock-in mice, which encode a gene that harbors the HT gene at the 5′ terminus of the talin1 gene to label talin1 endogenously by HT ligands (Fig. 1*B*) (15), and developed a system to measure *in vivo* single-molecule binding kinetics between talin1 and integrins using a modified total internal reflection microscopy system, which can monitor talin1 binding to integrin-CT in response to integrin–ligand interaction (Fig. 1, *B* and *C* and Movie S1) (6, 12). Single-molecule particle tracking (SPT) analysis revealed that talin1 binding frequency (event number of trajectories divided by the area of cell adhesion, Figure 1*D*, see the details in Experimental procedures section) (6) and talin1 binding duration (Fig. 1*E*) were significantly higher on integrin ligands than on poly-l-lysine (PLL), which does not bind specifically to integrins, indicating the integrin ligand binding–induced talin1 binding to respective integrins. Compared with β7–talin1 binding under MAdCAM1 conditions, β2–talin1 binding under ICAM1 conditions showed greater binding frequency (Fig. 1*D*) and a tendency for longer binding time (Fig. 1*E*) of talin1. Enhancement of α4/β7 expression by TCR–CD28 stimulation and retinoic acid supplementation (Fig. 1*F*) (19), which upregulated specific adhesiveness of α4/β7 to MAdCAM1 (Fig. 1, *G* and *H*), also resulted in greater talin1 binding frequency and time with β2 than with β7 (Fig. 1, *I* and *J*). Comparison of the dissociation rate constants (20) also showed that the binding time between β2 and talin1 was longer than that between β7 and talin1 in both naïve and activated T cells (Fig. 1*K*). These results suggested that talin1 binds more tightly to LFA1 than to α4/β7.

### Chimeric integrins to determine the net effect of the integrin CT on talin1 binding

**Figure 2:**
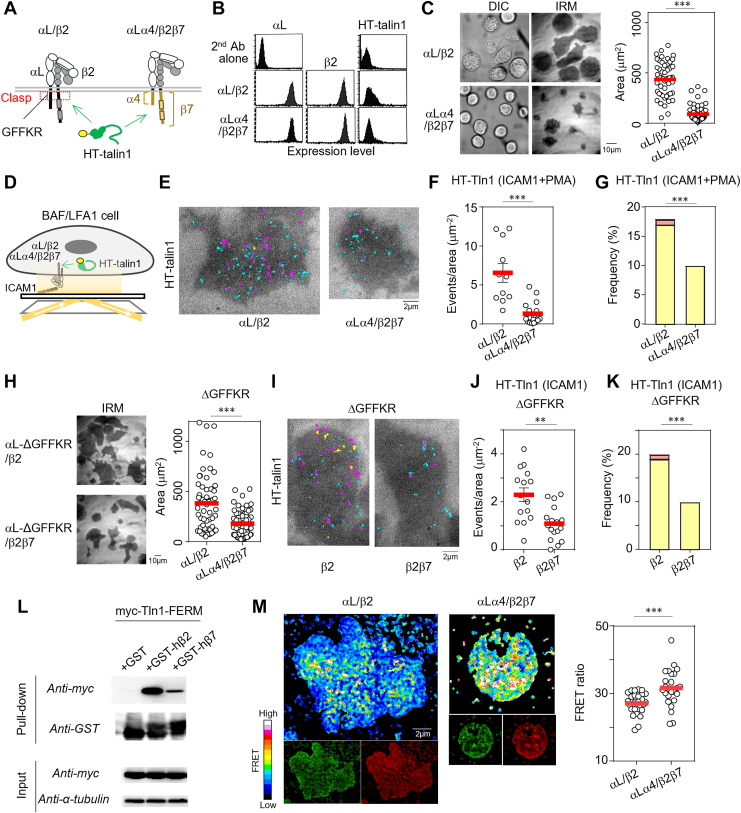
**Analyses of chimeric LFA1 harboring an α4/β7 cytoplasmic tail (CT)**. *A*, design of chimeric integrins. The CTs of αL and β2 were replaced with those of α4 and β7, respectively. *B*, surface expression of intact LFA1 and chimeric LFA1 on BAF/LFA1. *C*, adhesion assay. Areas of adhesion on hICAM1-coated glass-bottomed dishes with PMA stimulation were measured (αL/β2: *n* = 52, αLα4/β2β7: *n* = 68). A representative of more than three independent experiments is shown. *D*, schematic representation of *in vivo* HT-talin1 binding assay. The binding of HT-talin1 to the CT of intact or chimeric integrins was measured. *E*, single-molecule trajectories of talin1 in BAF/LFA1. Trajectories with binding duration <1 s, 1 to 10 s, and >10 s are colored *cyan*, *magenta*, and *yellow*, respectively. *F*, binding frequency (αL/β2: *n* = 11; αLα4/β2β7: *n* = 20) and *G*, binding duration of talin1 calculated as in Figure 1*E* (αL/β2: *n* = 4104; αLα4/β2β7: *n* = 971). Frequencies of 1 to 10 s and >10 s categories are shown in *yellow* and *red*, respectively. *H*, adhesion assay of BAF/LFA1 expressing αL-ΔGFFKR and intact β2 (*n* = 56) or β2β7 (*n* = 59) on hICAM1-coated glass-bottomed dishes in the absence of PMA. A representative of more than two independent experiments is shown. *I*, single-molecule trajectories of talin1 in αL-ΔGFFKR–expressing cells. Trajectories are shown as in (*E*). *J*, binding frequency (αL-ΔGFFKR/β2: *n* = 15; αL-ΔGFFKR/β2β7: *n* = 17) and *K*, binding duration of talin (αL-ΔGFFKR/β2: *n* = 1338; αL-ΔGFFKR/β2β7: *n* = 907) in αL-ΔGFFKR–expressing cells. Frequencies of binding time are shown as in (*G*). *L*, pull-down assay between the GST-fused integrin-CT (human β2 or β7) and myc-tagged talin1 FERM domain. More than two independent experiments were performed. *M*, intramolecular FRET analysis to monitor tension applied to talin1. A talin tension FRET sensor (Talin-FL-447) was used to visualize talin1 tension in cells (23) (αL/β2: *n* = 30; αLα4/β2β7: *n* = 25). Statistical analyses were performed with the unpaired nonparametric two-sided Student’s *t* test (*C*, *F*, *H*, *J*, and *M*) or Chi-squared test (*G*, *K*). ∗*p* < 0.01, ∗∗∗*p* < 0.001. FERM, four-point-one, ezrin, radixin, moesin; FL, ferredoxin-like; GST, glutathione-*S*-transferase; hICAM1, human intercellular adhesion molecule 1; PMA, phorbol 12-myristate 13-acetate.

**Fig S1:**
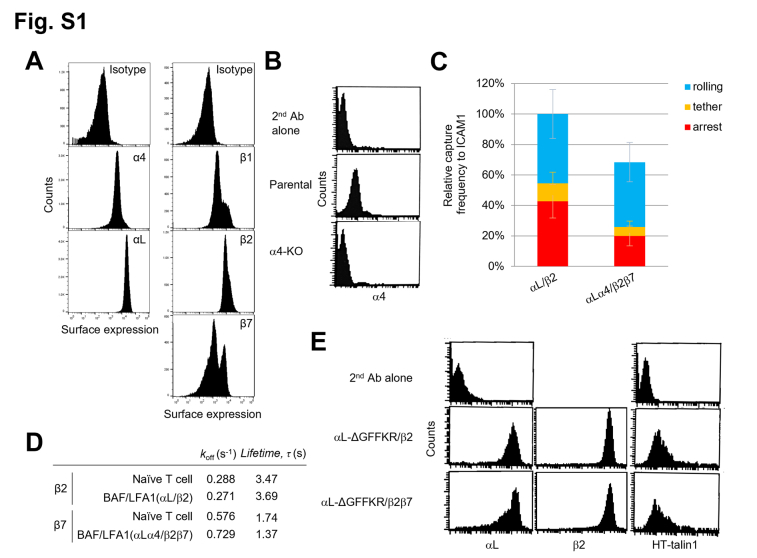


Despite a tendency toward adhesiveness and a talin binding hierarchy, with LFA1>α4/β7, the differences in their ligand types and expression levels hamper direct comparison (Figs. 1*F* and S1*A*). To precisely dissect the effects of differences in the integrin-CT on talin1 binding, we constructed chimeric integrins of LFA1 with α4 and β7 amino acid sequences in the CTs of αL and β2, respectively, for synchronization of ligand types (Fig. 2*A*). Utilizing the pro-B cell line Ba/F3, which lacks endogenous β1, β2, β3, and β7, but exclusively expresses HT-talin1 and is used as a model in studies of lymphocyte adhesion, we further deleted endogenous α4 (Fig. S1*B*) to avoid the unfavorable effects of its endogenous expression and expressed intact αL/β2 or chimeric αLα4/β2β7. We verified similar surface expression of intact αL/β2 and chimeric αLα4/β2β7 in the established cells (BAF/LFA1) (Fig. 2*B*). An adhesion assay for ICAM1 under phorbol 12-myristate 13-acetate (PMA) stimulation revealed weaker adhesion to ICAM1 of lymphocytes expressing αLα4/β2β7 compared with those expressing αL/β2 (Fig. 2*C*). SPT analyses of talin1 binding to the CT of αL/β2 or αLα4/β2β7 (Fig. 2, *D*, and *E*) revealed that αLα4/β2β7 had reduced binding frequency (Fig. 2*F*), binding time (Fig. 2*G*), and increased off-rate (Fig. S1*D*) to talin1 compared with αL/β2. Attenuation of cell adhesiveness of αLα4/β2β7 under shear flow conditions was also verified (Fig. S1*C*). As the chimeric mutation in αLα4/β2β7 may have led to structural perturbations around the inhibitory clasp region at the CT (Fig. 2*A*), which maintains the integrin in an inactive, bent-closed conformation (21), a GFFKR deletion mutant (ΔGFFKR) for disruption of the clasp to constitutively open the clasp to allow LFA1 to adopt an active conformation (Fig. 2*A*) (6, 22) was constructed (Fig. S1*E*). The αL-ΔGFFKR/β2 mutant showed spontaneous adhesiveness even in the absence of PMA, which is consistent with the previous report (Fig. 2*H*) (6). Although αL-ΔGFFKR/β2β7 also showed spontaneous binding to ICAM1 without PMA, its adhesion was weaker than that of αL-ΔGFFKR/β2 (Fig. 2*H*). SPT analysis of talin1 binding using the ΔGFFKR mutant (Fig. 2*I*) revealed decreased binding frequency (Fig. 2*J*) and time (Fig. 2*K*) for αL-ΔGFFKR/β2β7 compared with those of αL-ΔGFFKR/β2. These results suggested that β7-CT had weaker binding affinity for talin1 *in vivo* and induced less lymphocyte adhesiveness than β2-CT did.

### Biophysical properties of the binding of the β2 and β7 tails to talin1

To ascertain whether the difference in talin1 binding kinetics between β2 and β7 *in vivo* is attributable to differences in their binding properties, we generated glutathione-*S*-transferase (GST)-fusion constructs with β2-CT and β7-CT and assessed their ability to bind to talin1. Pull-down assays revealed that the talin1–FERM domain bound more strongly to GST-β2-CT than to GST-β7-CT (Fig. 2*L*).

### Comparison of forces imposed on talin1 between β2 and β7

**Fig S2:**
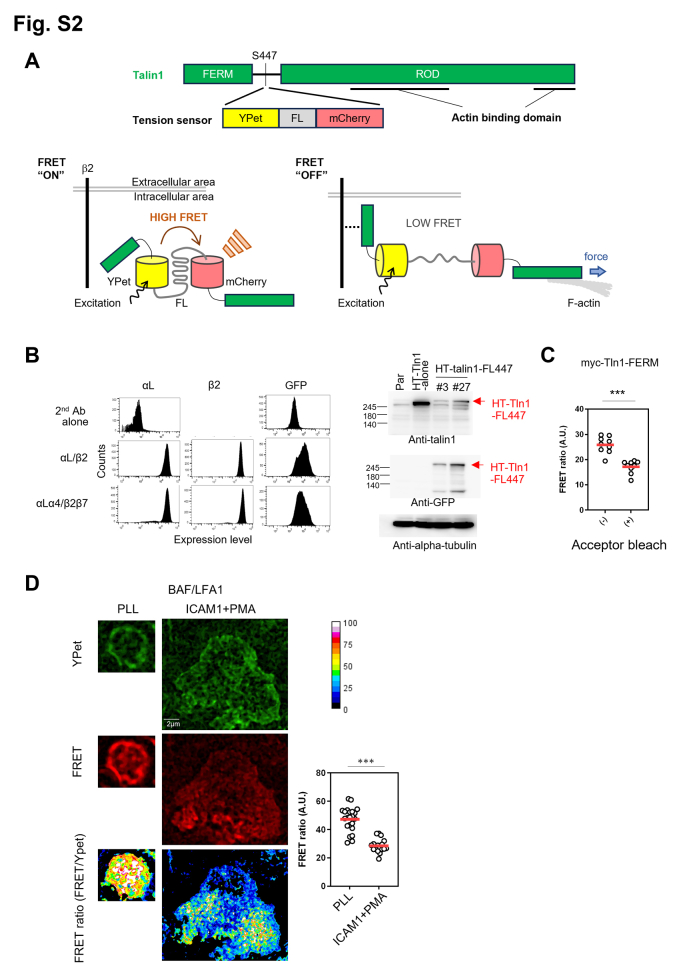


To investigate the cellular output according to the distinct binding kinetics between β2 and β7, we established lymphocytes harboring a talin1 tension sensor retaining a tension-sensitive ferredoxin-like (FL) linker peptide sequence flanked by YPet and mCherry (Fig. S2, *A*–*C*) (23). We first verified the tension sensor system in lymphocytes by comparing FRET signals near the contact surface in cells under conditions without (PLL) and with (ICAM1 + PMA) ICAM1-dependent adhesion (Fig. S2*D*). Compared with PLL conditions, ICAM1 + PMA conditions resulted in an attenuated FRET ratio, suggesting that ICAM1-dependent adhesion applies force to talin1, which is consistent with the general phenotype of integrin- and talin1-dependent cell adhesion (23). The αL/β2-expressing lymphocytes had weaker FRET signals than those expressing αLα4/β2β7 (Fig. 2*M*), verifying the functional integrity of talin in this system, and suggesting that β7 applies a weaker force to talin1 than β2 because of its lower binding affinity.

### New motif governing differences in talin1 binding to integrins

**Figure 3:**
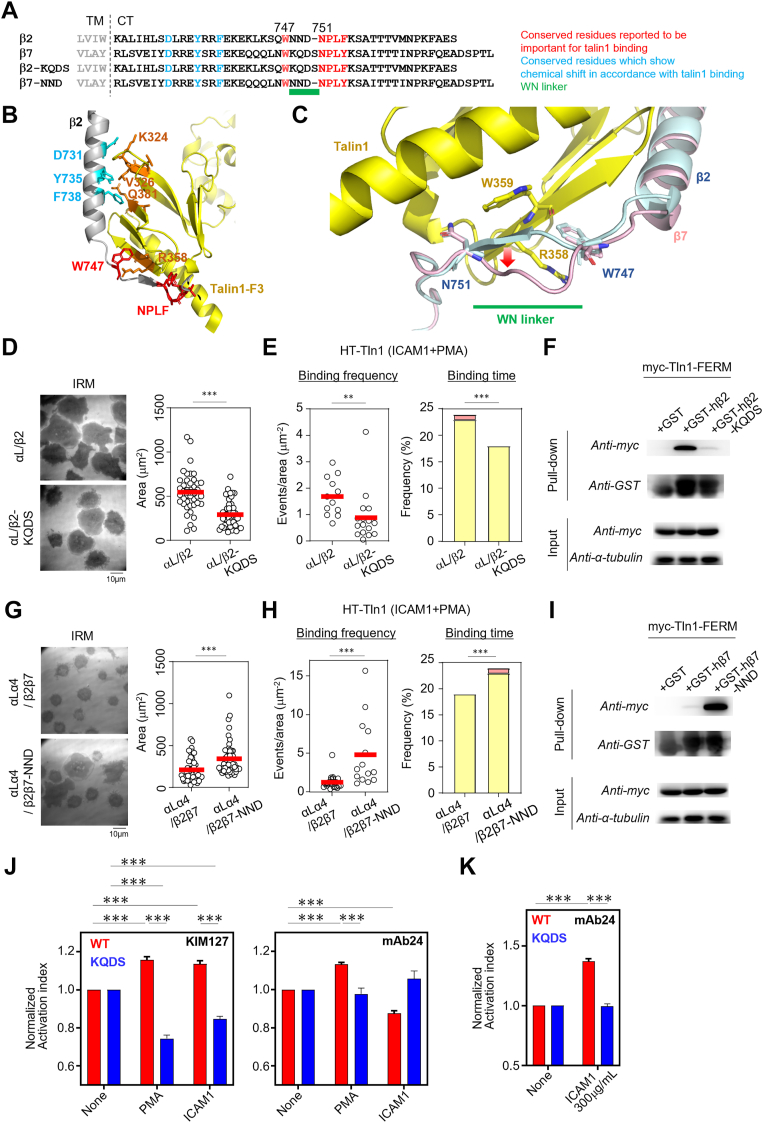
**Identification of the WN linker as a determinant of difference in talin1 binding between β2 and β7.***A*, comparison of the CT amino acid sequence between β2 and β7. The linker region between the conserved Trp747 (*red*) and conserved Asn751 (*red*) in β2 was named the WN linker (*green underline*). *Cyan-highlighted residues* indicate conserved residues located within 7 Å (Cα–Cα distance) of talin residues exhibiting NMR chemical shifts upon integrin binding (24). *B*, model structure of β2–talin1 complex predicted by AlphaFold2. The residues in β2 shown in *cyan* and in talin1 shown in *red* correspond to the residues shown in the same color in (*A*). Residues with changes in NMR chemical shifts in accordance with β2 binding are shown in *orange* (24). *C*, difference between β2 (*blue*) and β7 (*pink*) in the binding mode of talin1 (*yellow*) in the AlphaFold2-predicted model. *Red arrow*: kink in β7 compared with β2. *D*, effects of replacement of the WN linker on lymphocyte adhesion. The NND sequence in the β2 WN linker was replaced with the KQDS sequence from the β7 WN linker. This mutant (β2-KQDS, *n* = 47) was compared with intact β2 (*n* = 42). A representative of more than two independent experiments is shown. *E*, single-molecule binding analysis of talin1 to integrin-CT in β2- or β2-KQDS-expressing BAF/LFA1. Binding frequency (*left*, β2: *n* = 12; β2-KQDS: *n* = 16) and binding duration (*right*, β2: *n* = 2294; β2-KQDS: *n* = 1315) of talin1 are shown. Frequencies of 1 to 10 s and >10 s categories are shown in *yellow* and *red*, respectively. *F*, pull-down assay between GST-fused integrin-CT (human β2 or β2-KQDS) and myc-tagged talin1 FERM domain. More than two independent experiments were performed. *G*–*I*, effects of replacement of KQDS in the β7 WN linker with NND on lymphocyte adhesion (*G*, β2β7: *n* = 49; β2β7-NND: *n* = 53), single-molecule talin1 binding kinetics (*H*, *left*, β2β7: *n* = 22; β2β7-NND: *n* = 14; *right*, β2β7: *n* = 1453; β2β7-NND: *n* = 1726, frequencies of binding time are shown as in (*E*)), and *I*, pull-down assay of GST-fused integrin and talin1 FERM domain. More than two independent experiments were performed. *J*, effects of the WN linker mutation on LFA1 activation. Normalized activation indices were compared between BAF/LFA1 cells expressing αL/β2 (WT) and those expressing αL/β2-KQDS (KQDS). (*Left*) KIM127 and (*Right*) mAb24. Conditions were as follows: None, no stimulation; PMA, 100 ng/ml PMA; and ICAM1, 3 μg/ml human ICAM1 (a concentration sufficient to induce LFA1-dependent cell adhesion). *n* = 4. *K*, effect of the WN linker mutation for the formation of extended-open conformation at the extremely high concentration of ICAM1 (300 μg/ml human ICAM1). *n* = 4. Statistical analyses were performed using the unpaired nonparametric two-sided Student’s *t* test (*D*, *E*, *G*, and *H*) or Chi-squared test (*E*, *H*) or two-way ANOVA with Tukey’s correction for multiple comparisons (*J*, *K*). ∗*p* < 0.05, ∗∗*p* < 0.01, and ∗∗∗*p* < 0.001. CT, cytoplasmic tail; FERM, four-point-one, ezrin, radixin, moesin; GST, glutathione-*S*-transferase; ICAM1, intercellular adhesion molecule 1; PMA, phorbol 12-myristate 13-acetate.

To delineate the region responsible for the differences in talin1 binding kinetics between β2 and β7 integrins, we compared their CT sequences (Fig. 3*A*). Key residues critical for talin1 regulation, including W747 and N751–P752–L753–F754 in β2 (7, 12), are highly conserved in β7 (Fig. 3*A*). Similarly, residues forming the talin1-binding interface (Fig. 3*B*), such as Y735 and F738, which induce a chemical shift change in NMR after talin1 binding (24), were also conserved (Fig. 3, *A* and *B*). Given these similarities, we focused on the linker region between W747 and N751–P752–L753–F754 in β2, which we named the WN linker (Fig. 3*A*).

Structural models of the β2-CT–talin1 and β7-CT–talin1 complexes generated using AlphaFold2 closely resembled the β1D-CT–talin2 crystal structure (Protein Data Bank ID: 3G9W) (Fig. 3*C*) (25). Notably, the WN linker in β7-CT protruded outward from the integrin–talin1 interface, whereas in β2-CT, it formed a β-strand integrated into a β-sheet with talin1 (Fig. 3*C*).

**Fig S3:**
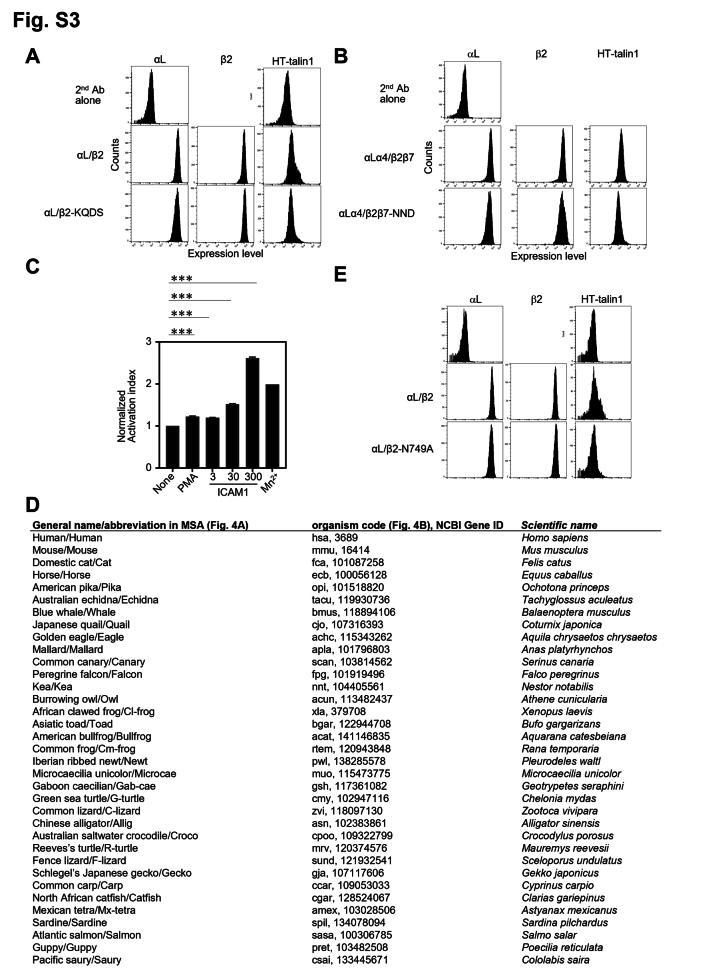


Based on these structural insights, we replaced the WN linker in β2-CT with the KQDS sequence from β7-CT (β2-KQDS, Fig. 3*A*). Lymphocytes expressing αL/β2-KQDS exhibited reduced ICAM1-dependent adhesion despite having similar LFA1 surface expression levels (Figs. 3*D* and S3*A*). The KQDS mutation reduced β2–talin1 binding frequency and duration *in vivo* (Fig. 3*E*). Consistently, pull-down assay confirmed weaker talin1 binding of β2-KQDS than WT β2 (Fig. 3*F*).

Conversely, replacing the WN linker in β7-CT with the NND sequence from β2-CT (β7-NND, Fig. 3*A*) increased adhesiveness of αLα4/β2β7 lymphocytes (Figs. 3*G* and S3*B*). Correspondingly, both *in vivo* and *in vitro* assays showed increased talin1 binding strength (Fig. 3, *H* and *I*). These results demonstrated that the WN linker is a critical determinant of the differential talin-binding kinetics of β2 and β7 integrins.

To investigate the role of the WN linker in regulating LFA1 conformational changes, we performed epitope staining assays using conformation-specific monoclonal antibodies, KIM127, which recognizes the extended active conformation of the β2 integrin subunit, and monoclonal antibody 24 (mAb24), which recognizes the extended-open (EO) conformation, a high-affinity conformation for ICAM1, of the β2 integrin (6). Upon inside–out stimulation with PMA, lymphocytes expressing αL/β2 (WT) exhibited a clear induction of the active conformation recognized by both KIM127 and mAb24, whereas cells expressing αL/β2-KQDS showed no active conformational change (Fig. 3*J*), suggesting that the β2-derived WN linker is important for talin1-mediated activation of LFA1. We next assessed whether the WN linker also influences outside–in signaling, specifically the ligand-induced conformational changes recently reported in α4/β1 and α5/β1 integrins (16). First, we verified that ligand engagement can indeed induce conformational changes also in LFA1 (Fig. S3*C*). We then found that the replacement of the WN linker similarly impaired ligand-induced extension of LFA1 (Fig. 3*J*, *left*), whereas ligand binding alone did not induce conformational change sufficient to expose the mAb24 epitope (Fig. 3*J*, *right*). However, the addition of an extremely high concentration of ICAM1 induced an mAb24 epitope exposure, which was inhibited by the replacement of the WN linker (Fig. 3*K*). These results suggest that the WN linker contributes to LFA1 conformational activation through its modulation of talin1 binding strength, which is induced both by inside–out and by outside–in stimulation.

**Figure 4:**
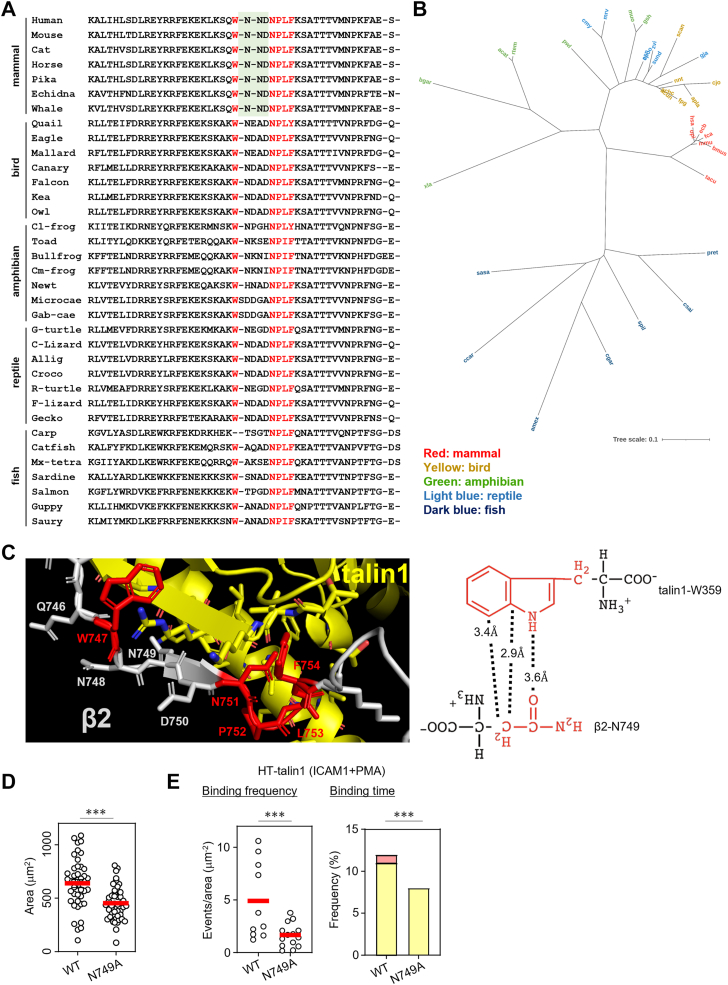
**Features of mammalian β2-CT sequence**. *A*, multiple sequence alignment (MSA) of CT region of vertebrate β2 integrins (seven sequences in each clade). Species from different orders are selected as much as possible. Conserved residues (W) and motifs (NPxY/F) essential for integrin–talin1 interaction are highlighted in *red*. The NND sequence, conserved only in mammalian β2 integrins, is shaded in *green*. Species used in MSA are listed in Fig. S3*D*. *B*, unrooted phylogenetic tree based on the MSA of amino acid sequences. Amino acid sequences were aligned using MAFFT (https://www.genome.jp/tools-bin/mafft) with the G-INS-i strategy, employing the BLOSUM62 scoring matrix, a gap opening penalty of 1.0, and an offset value of 0.14. The resulting alignment was used to construct an unrooted phylogenetic tree with branch lengths using the neighbor-joining method. Organism codes are shown. Tree visualization was refined using iTOL (https://itol.embl.de/). *C*, *left*, close-up view of the talin1–β2-CT structure. Talin1 and β2 are shown in *yellow* and *gray*, respectively. The conserved W747 and the NPxF motif are highlighted in *red*. While the side chains of N748 and D750 face away from talin1, the side chain of N749 is positioned in close proximity to talin1. *Right*, distances between the side chain of N749 in β2-CT and W359 in talin1 are indicated. *D*, effects of N749A mutation on lymphocyte adhesion. WT: *n* = 42, N749A: *n* = 48. *E*, single-molecule binding analysis of talin1 to integrin-CT in WT β2– or β2-N749A–expressing BAF/LFA1. Binding frequency (*left*, WT: *n* = 10; N749A: *n* = 14) and binding duration (*right*, WT: *n* = 4458; N749A: *n* = 2295) of talin1 are shown. Frequencies of 1 to 10 s and >10 s categories are shown in *yellow* and *red*, respectively. Statistical analyses were performed using the unpaired nonparametric two-sided Student’s *t* test (*D*, *E*) or Chi-squared test (*E*). ∗∗∗*p* < 0.001. CT, cytoplasmic tail.

**Figure 5:**
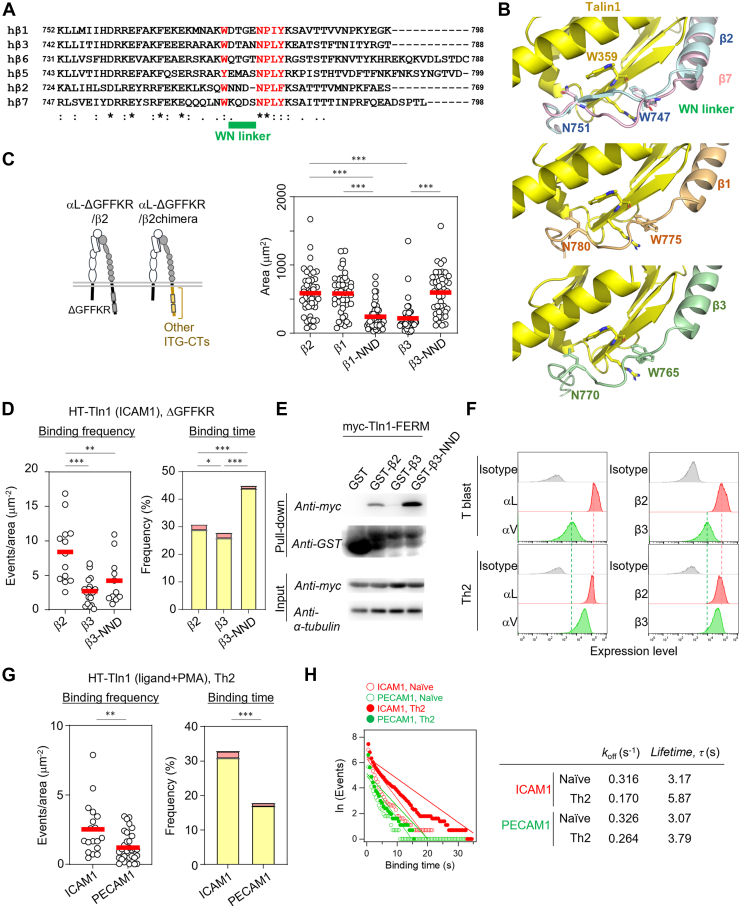
**Importance of the WN linker in other integrins.***A*, comparison of CT amino acid sequence among talin1-binding integrins. The WN linker region and the conserved Trp and NPxY/F motif are highlighted as in Figure 3. *B*, model structure of β1– or β3–talin1 complex. The conserved Trp and NPxY/F motif in each integrin are labeled. Colors for talin1 and integrins are as in Figure 3, except those for β1a (*orange*) and β3 (*green*). *C*, adhesion of αL-ΔGFFKR-expressing BAF/LFA1 onto ICAM1-coated dishes in the absence of PMA. The indicated β integrins were also expressed in BAF–LFA1. β2 (*n* = 46), β1 (*n* = 42), β1-NND (*n* = 47), β3 (*n* = 42), and β3-NND (*n* = 45). *D*, single-molecule binding analysis of talin1 to integrin-CT in β2-, β2β3-, or β2β3-NND-expressing BAF/LFA1. The binding frequency (*left*) and binding duration (*right*) of talin1 are shown. Frequencies of 1 to 10 s and >10 s categories are shown in *yellow* and *red*, respectively. *E*, pull-down assay between GST-fused integrin-CT (human β2-, β3-, or β3-NND) and myc-tagged talin1 FERM domain. More than two independent experiments were performed. *F*, integrin expression profile of Th2-skewed T-cell blasts compared with conventional T-cell blasts. *G*, single-molecule binding analysis of talin1 in Th2 cells. *Left*, binding frequency (ICAM1: *n* = 18; PECAM1: *n* = 32). *Right*, binding duration (ICAM1: *n* = 1724; PECAM1: *n* = 733). Frequencies of binding time are shown as in (*D*). *H*, the dissociation rate constant of talin1 to β2 or β3, *k*_off_, was calculated as in Figure 1*K*. Statistical analyses were performed by one-way ANOVA with Tukey’s correction for multiple comparisons (*C* and *D*), unpaired nonparametric two-sided Student’s *t* test (*G*, *left*), or Chi-squared test for trend (*D* and *G*, *right*). ∗*p* < 0.05, ∗∗*p* < 0.01, and ∗∗∗*p* < 0.001. CT, cytoplasmic tail; FERM, four-point-one, ezrin, radixin, moesin; GST, glutathione-*S*-transferase; ICAM1, intercellular adhesion molecule 1; NPxY/F, Asn-Pro-x-Tyr/Phe; PECAM1, platelet endothelial cell adhesion molecule 1; PMA, phorbol 12-myristate 13-acetate.

Multiple sequence alignment analyses of the integrin β-CT regions revealed that the three-residue NND sequence is unique to β2 integrins and is largely restricted to mammals (Figs. 4, *A* and *B* and 5*A*). Notably, the second asparagine in the NND sequence (N749 in β2) is highly conserved among β2 orthologs in mammals and is not found in other integrin CTs. Structural modeling of the talin1–β2-CT complex showed that the side chain of N749 is positioned in close proximity to talin1, suggesting a potential hydrogen bond and Van der Waals interaction with the talin1 residue W359 (Fig. 4*C*). In contrast, the neighboring residues N748 and D750 are oriented away from the talin1 surface (Fig. 4*C*). To investigate the functional relevance of N749, we generated BAF/LFA1 cells expressing the β2-N749A mutant (Fig. S3*E*). Compared with WT-β2, the N749A mutant exhibited significantly reduced adhesion to ICAM1 (Fig. 4*D*) and impaired talin1 binding, as reflected by both decreased binding frequency and shorter binding duration in single-molecule assays (Fig. 4*E*). Taken together, these findings indicate that the NND motif, especially N749, plays a critical role in stabilizing the talin1–β2 interaction, thereby promoting integrin activation and lymphocyte adhesion.

### Functional role of the WN linker in other integrins

**Fig S4:**
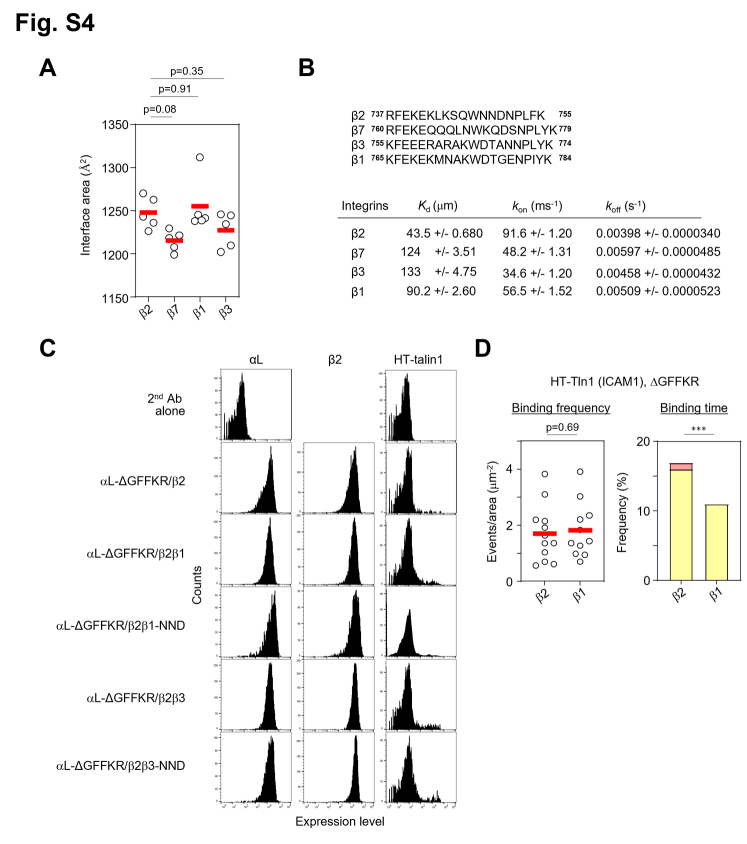


To assess the broader relevance of WN linker–mediated integrin regulation, we analyzed the β-CT sequences of β1, β2, β3, β5, β6, and β7 (Fig. 5*A*), excluding β4 and β8 that lack the canonical talin1 binding motif (3). All integrins examined, except β2, contained four-residue WN linkers. Structural models of the β1-CT–talin1 and β3-CT–talin1 complexes implicated that these integrins adopted WN linker conformations similar to β7-CT (Figs. 3*C* and 5*B*). On the other hand, PISA (Proteins, Interfaces, Structures, and Assemblies) (26) analysis implied that the average interface area calculated from five model structures of the β2-CT–talin1 complex was likely to be larger than that of the β7-CT–talin1 complex and the β3-CT–talin1 complex; however, the average interface area of the β2-CT–talin1 complex was comparable to or slightly smaller than that of the β1-CT–talin1 complex (Fig. S4*A*). We also conducted an *in vitro* quantitative binding assay between synthesized β integrin-CT peptides and the purified talin1 FERM domain using Octet biolayer interferometry measurement system (27) and found that talin1 binds to β2 integrin with threefold higher affinity than β7 and β3 and with twofold higher affinity than β1 (Fig. S4*B*), partially supporting the structural model in terms of the order of binding strength of β integrin-CT to talin1, and *in vitro* pull-down assay results, whereas the order of the difference *in vivo* is still elusive.

To examine talin1–β integrin-CT binding properties and their functional features *in vivo*, we generated BAF/LFA1 cells expressing chimeric αL-ΔGFFKR/β2β1 or αL-ΔGFFKR/β2β3 integrins (Figs. 5*C*, *left* and S4*C*). αL-ΔGFFKR/β2 and αL-ΔGFFKR/β2β1 exhibited similar adhesiveness, whereas αL-ΔGFFKR/β2β3 showed reduced adhesion (Fig. 5*C*, *right*). Furthermore, replacement of the WN linker in β3-CT with NND (β3-NND) (Fig. S4*C*) increased adhesion in αL-ΔGFFKR/β2β3 cells (Fig. 5*C*, *right*), suggesting that the WN linker in β3, like β7, exerts an inhibitory effect on integrin adhesiveness. In contrast, replacement of the WN linker in β1-CT with NND (β1-NND) reduced adhesion (Fig. 5*C*, *right*). The SPT assay of talin1 demonstrated that the *in vivo* binding frequency of talin1 to β1-CT is comparable to that of β2-CT, although the binding time is shorter for β1-CT (Fig. S4*D*). Together with the PISA analysis (Fig. S4*A*) and Octet binding assays (Fig. S4*B*), these results suggest that the WN linker in β1-CT binds to talin1 slightly more weakly than that in β2-CT but retains distinct structural features compared with those in β2-, β3-, β7-CTs.

SPT assay of talin1 for β3 binding revealed reduced binding frequency and duration for β3-CT compared with β2-CT, which was increased by NND substitution (Fig. 5*D*). Pull-down assay confirmed that β3 bound talin1 less effectively than β2, whereas WN linker replacement increased talin1 binding (Fig. 5*E*). These findings underscore that the WN linker proximity to talin1 in both β2 and β3 is a determinant of the difference in integrin–talin1 binding affinity.

### Natural ligand-induced talin binding to β3 integrins in T cells

To examine integrin–talin1 interactions mediated by intact β3 integrins, we assessed the kinetics of talin1 binding to β3 integrins in primary T cells. Among the β3-associated integrins, αIIb/β3 and αv/β3, αv/β3 expression in T cells was hypothesized based on the ImmGen database (Fig. S5*A*) and confirmed by fluorescence-activated cell sorting analysis (Fig. S5*B*). Using platelet endothelial cell adhesion molecule 1 (PECAM1) as a specific ligand (28), we observed dose-dependent T-cell adhesion to PECAM1-coated surfaces, albeit weaker than that to ICAM1 (Fig. S5*C*). *In vivo* single-molecule talin1 binding assay revealed significantly weaker talin1 binding to αV/β3 than to αL/β2 in naïve T cells (Fig. S5*D*). Enhancement of αV/β3 integrin expression by skewing to the Th2 subset (Fig. 5*F*) (29), which induced elevated adhesion to PECAM1 dependent on β3 (Fig. S5, *E* and *F*), also revealed the same tendency of talin1 binding kinetics (Fig. 5*G*). The off-rate of talin1 to β3 was also higher than that to β2 (Fig. 5*H*). These findings suggest that β3 integrin also has an inhibitory-type WN linker with weaker talin1 binding ability than β2 integrin does.

## Discussion

**Figure 6:**
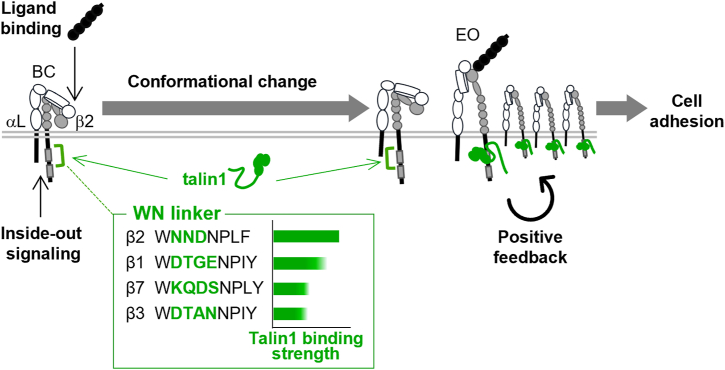
**Updated model of LFA1 activation.** Our earlier work demonstrated that ligand engagement and outside–in signaling promote talin1 binding to LFA1 more frequently and with longer dwell times than inside–out signaling alone (6). Following ligand binding and the associated conformational change, a positive feedback loop is triggered, driving further talin1 recruitment and LFA1 accumulation at the contact interface. Inside–out signaling functions as a prerequisite “fire starter” to initiate this feedback activation, but the molecular details of the earliest steps in this process were previously unclear. In this study, we found that both inside–out signaling and ligand engagement can initiate the activatory conformational changes in LFA1 through talin1 binding to the WN linker region (Fig. 3, *E* and *J*). This interaction facilitates the transition from the bent-closed (BC) conformation to the extended-open (EO) conformation. Once in the EO state, LFA1 binds tightly to ICAM1, reinforcing positive feedback activation. Notably, a concentration of ICAM1 (3–10 μg/ml) that is sufficient to support inside–out-dependent adhesion cannot promote adhesion in the absence of inside–out signaling (6). However, higher ICAM1 concentrations, which more efficiently promote the active conformation (Figs. 3*K* and S3*C*), could partially bypass the requirement for “fire starter” inside–out signaling and support adhesion through a ligand-driven pathway. Importantly, during these talin1-dependent steps, the WN linker plays a critical role by modulating talin1 binding strength across different integrin subtypes. ICAM1, intercellular adhesion molecule 1.

We identified a previously uncharacterized talin1-binding region within the β integrin-CT region, named the WN linker, using an *in vivo* single-molecule integrin–talin1-CT binding assay. While most integrin-CTs contain a four-residue WN linker, β2-CT uniquely possesses a three-residue variant (NND). Substituting the NND sequence in β2-CT with the four-residue WN linker from β3 or β7 significantly weakened talin1 binding and reduced lymphocyte adhesiveness. In contrast, replacing the four-residue WN linker in β3- or β7-CT with NND enhanced talin1 binding and increased adhesiveness. The critical role of the second asparagine (N749), which is highly conserved within the NND sequence, in mediating talin1 binding and promoting lymphocyte adhesiveness further supports this notion. These findings establish the WN linker as a critical determinant of integrin–talin1 interaction, directly regulating cell adhesion (Fig. 6).

The discrepancy in the order of talin1 binding strength for integrins between the previous study (β7 > β2, β3) (4) and this report (β2 > β3, β7) may be due to differences in sequences other than integrin-CTs. In our case, we used endogenous integrin-CTs *in vivo* or integrin-CTs with the linker sequence from pGEX-4T2 for the pull-down assay. In the previous study, a sequence that induced a coiled-coil formation, four heptad-repeats (30), was added to the N terminus of integrin-CTs, and this difference in three-dimensional structure may have been responsible for the differences.

Interestingly, substituting the β2-CT WN linker with that of β1-CT did not diminish cell adhesiveness. Instead, replacing the β1-CT WN linker with NND reduced adhesion. Previous reports have shown that the β1-CT, including its WN linker, is highly conserved across vertebrates (31), raising the possibility that the β1-CT sequence underwent evolutionary optimization early in vertebrate evolution. In our present work, PISA analysis revealed that the interface area of β1-talin1 is comparable to that of β2–talin1. Consistently, both our *in vitro* Octet analysis (Fig. S4*B*) and *in vivo* single-molecule binding assays (Fig. S4*D*) demonstrated that β1-CT binds to talin1 slightly less strongly than β2-CT does but more strongly than β3-CT or β7-CT. Taken together, these results suggest that the β1-CT possesses structural features distinct from β2-, β3-, and β7-CTs in terms of talin1 binding.

In contrast, our multiple sequence alignment analysis of β2-CTs revealed that the NND motif is conserved exclusively in mammals (Fig. 4*A*). Nonmammalian vertebrate β2-CTs instead contain a four- or five-residue WN linker similar to other integrins (Fig. 4*A*). Furthermore, phylogenetic analysis revealed that mammalian β2-CT sequences form a distinct cluster with limited sequence diversity compared with those of other vertebrates (Fig. 4*B*). Although the clear reason for the complete conservation of NND sequence and limited diversity of β2-CT in mammals is still unclear, this may reflect a finely tuned leukocyte/lymphocyte-mediated immune system in mammals, requiring immediate and potent activation of integrin-mediated adhesion in response to a plenty of pathogen invasions.

The identification of a novel talin1 binding motif, the WN linker, together with evidence demonstrating the importance of both inside–out signaling and ligand binding in promoting talin1-dependent LFA1 conformational changes (Fig. 6), is consistent with our previous findings. We previously showed that the binding kinetics of ICAM1 and talin1 to LFA1 are almost identical, suggesting that simultaneous occupancy by ICAM1 and talin1 is required for LFA1-mediated adhesive responses, as each binding depends on the other (6). In line with this, ICAM1-mediated slow rolling occurs concurrently with talin1 recruitment (15). Thus, talin1 likely contributes to the initial stage of conformational changes of bent-closed LFA1, enabling slow rolling, and promotes transition to the high-affinity/EO conformation that mediates firm arrest (Fig. 6). Once engaged with both ICAM1 and talin1, the high-affinity/EO LFA1 triggers a positive feedback loop of activation through robust outside–in signaling involving Rap1 and talin1 (Fig. 6). Because high-affinity binding of LFA1 requires an actin cytoskeleton and forces transmission *via* talin1 (6), the presence of the WN linker may help stabilize talin1 and β2 CT interactions. Moreover, since mutations of the WN linker substantially reduced the frequency and duration of talin1 binding, it might be required from the early stage of conformational change. However, the precise action point of the WN linker—either in enabling slow rolling or the subsequent arrest and strengthening of adhesion—remains unclear at present. In contrast, α4/β7 and MAdCAM1 can mediate the rolling and tethering of lymphocytes in the absence of Rap1 and talin1, although arrest adhesion still requires both (15). Thus, transient ligand interactions and rolling of α4/β7 (and perhaps also rolling-supportive α4/β1) do not require talin1 or Rap1, in agreement with a ligand-dominant role in initiating integrin conformational changes of α4 and α5 integrins (16). Thus, bidirectional integrin activation varies among integrin species.

The WN linker has clinical therapeutic potential. The human gene encoding β2 integrin, *ITGB2*, has been implicated in leukocyte adhesion deficiency I (LAD-I) (32), a disorder characterized by defective immune cell adhesion. While numerous LAD-1–associated mutations have been identified, none have been reported within the WN linker region (ClinVar: https://www.ncbi.nlm.nih.gov/clinvar/). Inversely, β2 integrin overexpression has been linked to inflammatory diseases, such as rheumatoid arthritis, osteoarthritis, and systemic sclerosis (33, 34, 35, 36). Targeting the WN linker with inhibitory peptides or small molecules could mitigate pathological integrin activation in these diseases while minimizing the risk of LAD-1, thus opening novel therapeutic avenues.

## Experimental procedures

### Mice

All protocols used in animal experiments were approved by the Animal Care and Use Committee of Kansai Medical University (approval no.: 24-058). The mice used in this study were maintained under specific pathogen-free conditions in the animal facility of Kansai Medical University. C57BL/6 mice were obtained from CLEA Japan, Inc. HT-talin1 knock-in mice were generated as described previously (15). Mice aged 8 to 20 weeks were used for the preparation of CD3^+^ T cells.

### Reagents and antibodies

HT SaraFluor 650T Ligand was purchased from Goryo Chemical. PMA, anti-α-tubulin antibody, aprotinin, cOmplete ULTRA tablets, and RPMI1640 culture medium were from Sigma–Aldrich. Mouse MAdCAM1/Fc chimera was from R&D Systems. Glass-bottomed dishes, 35 mm in diameter, were from Matsunami. Phycoerythrin-conjugated isotype control, anti-mouse CD49d, anti-mouse CD51 antibodies, allophycocyanin-conjugated isotype control, anti-mouse CD11a/CD18, anti-mouse/rat CD61, anti-human/mouse integrin β7 antibodies, Alexa Fluor 647–conjugated anti-mouse/rat CD29 antibody, antipurified anti-mouse interferon-γ, and MojoSort Mouse CD3 T Cell Isolation Kit were from BioLegend. Anti-human IgG antibody was from Rockland. Anti-GST-Tag mouse mAb was from Cell Signaling Technology. Anti-mouse CD11a phycoerythrin antibody was from eBioscience. Recombinant human interleukin (IL)-2, recombinant murine IL-7, and recombinant murine IL-4 were from Peprotech. All-*trans*-retinoic acid is from Fujifilm-Wako. Mouse CD31/PECAM-1 protein was from MedChemExpress. The In-Fusion HD Cloning Kit was from Takara. The KOD-Plus-Mutagenesis Kit was from Toyobo. Anti-myc antibody (9E10), anti-human αL antibody (TS2/4), and anti-human β2 antibody (TS1/18) were purified from hybridomas purchased from the American Type Culture Collection.

### Plasmids and cells

Chimeric mutants of αL and β2 carrying the CT regions of other integrins, their mutants, and talin1 with FRET sensor were constructed by PCR and with an In-Fusion HD Cloning Kit and KOD-Plus-Mutagenesis Kit. The α4 integrin in BAF/LFA1, in which β1, β2, β3, and β7 had already been deleted and exclusively expressed HT-talin1 (6), was further deleted using guide RNA–encoding oligonucleotides: TTCCCGGAGGGCGATCCCTC and ATTACCATCAGCTTGCTACT, to prevent the effects of endogenous α4 integrin on adhesion and talin1 single-molecule assay. The established BAF/LFA1 with deletion of *Itga4* was used as a parental cell line for the expression of mutants of αL and β2. Mouse CD3^+^ T cells were purified from splenocytes by negative selection using a MojoSort Mouse CD3 T Cell Isolation Kit after removal of red blood cells by incubation for 1 min in ACK buffer. For the preparation of α4 integrin–enhanced T cells, purified CD3^+^ T cells (3 × 10^5^ cells) were stimulated on 10 μg/ml anti-CD3–coated wells of 96-well plates in Iscove’s modified Dulbecco’s medium supplemented with 4% fetal calf serum, 55 μM 2-mercaptoethanol, penicillin–streptomycin, and 4 μg/ml anti-CD28. After 2 days in culture, cells were transferred to uncoated wells, and 10 ng/ml IL-2 and 2 ng/ml IL-7 with or without 10 nM all-*trans*-retinoic acid were added. Cells were used for the assay after 2 to 3 days. For the preparation of αV integrin–enhanced T cells, CD3^+^ T cells (3 × 10^5^ cells) were stimulated on anti-CD3–coated wells as described previously in Iscove’s modified Dulbecco’s medium supplemented with 4% fetal calf serum, 55 μM 2-mercaptoethanol, P/S, 4 μg/ml anti-CD28, 20 ng/ml IL-2, 50 ng/ml IL-4, and 20 μg/ml anti-interferon-γ. After 2 days, cells were transferred to uncoated wells and cultured in the same medium without anti-CD28. Cells were used for analysis after a further 2 days in culture.

### Lymphocyte adhesion assay

For adhesion assay of BAF/LFA1, glass-bottomed dishes 35 mm in diameter (Matsunami) were coated with anti-human IgG antibody (15 μg/ml), washed with PBS containing 0.1% bovine serum albumin, and further coated with human ICAM1-Fc (3 μg/ml) (37). Cells were washed with IL3-free medium and plated on the coated dishes in the presence of 100 ng/ml PMA. In the case of BAF/LFA1 expressing αL-ΔGFFKR, the assay was performed in the absence of PMA. After application and incubation at 37°C for 15 to 45 min, differential interference contrast microscopy and interference reflection microscopy images were captured by MetaMorph (Molecular Devices) using an IX81 microscope (Olympus). The areas of interference reflection microscopy were measured by manually defining the area of adhesion with polygon selections in Fiji and subjected to statistical analysis with Prism (GraphPad Software). Adhesion assay under shear flow conditions was performed as described previously (15). Statistical analysis was performed using the Student’s *t* test or one-way ANOVA.

For adhesion assay of mouse primary T cells, glass-bottomed dishes were prepared as described previously (37) except for the ligand type: mouse ICAM1 (0.1–30 μg/ml), mouse MAdCAM1 (0.1–30 μg/ml), and mouse PECAM1 (0.1–10 μg/ml). Stimulation and image capture were performed as described previously (37).

### Single-molecule analysis of talin1 binding

*In vivo* single-molecule talin1 binding kinetics were measured as described previously (6). Briefly, HT-talin1 expressed in BAF/LFA1 or T cells from mice was labeled with HT SaraFluor 650T Ligand (<1 nM) for 1 h at 37 °C. The labeled cells were plated onto ligand-coated glass-bottomed dishes and imaged by total internal reflection microscopy as described (6). Individual spots were analyzed using the G-count and G-track software (G-angstrom) to generate trajectories (>0.2 s) (6). The obtained trajectories were analyzed using MATLAB, Prism, and Fiji as described previously (6, 20).

### Pull-down assay

GST pull-down assay was performed as described previously (6) using GST, GST-fusion constructs with β2-CT, β7-CT, and β3-CT, and these mutants (β2-KQDS, β7-NND, and β3-NND), as well as myc-tagged talin1 FERM domain. Integrin-CTs (Figs. 3*A* and 5*A*) were fused to the C terminus of GST with the linker sequence SDLVPRGSPGIPGSTRA. GST alone with the linker sequence and additional AAS sequence at the C terminus of the linker was used as a negative control. Lysates of 293T cells expressing myc-tagged talin1 FERM were mixed with Glutathione Sepharose beads (Cytiva), preimmobilized with GST or GST-integrin-CT, and rotated for 1 h at 4 °C. The beads were washed with lysis buffer (30 mM Tris–HCl, 150 mM NaCl, 1 mM EDTA, 0.15% Triton X-100, cOmplete ULTRA tablets [Roche], pH 7.5) and subjected to Western blotting. Signals were detected by chemiluminescence using Western Lightning Plus-ECL (Revvity Health Sciences).

### Flow cytometry

Surface integrin levels were measured by flow cytometry as described previously (6). Briefly, the cells were stained with TS2/4 or TS1/18 on ice for 30 min. The cells were further stained with anti-mouse eFluor 660 IgG. For primary T cells, antibodies for each integrin conjugated directly with dyes were used. The stained cells were analyzed by flow cytometry on a FACSCalibur or Attune 4 Nxt instrument. The obtained data were analyzed with FlowJo software (BD Science).

### FRET analysis

Ratiometric FRET analyses of live BAF/LFA1 cells expressing talin, with insertion of fusion protein (YPet-an FL linker peptide-mCherry) between S447 and G448 in the talin1 linker region (23) were performed to monitor the tension applied to talin using a Dragonfly dual-camera system equipped with a 100× oil-immersion objective. The YPet-FL-mCherry probe could monitor the tension applied to talin1 by tracing the decrease in FRET between YPet and mCherry. The entire area of cell adhesion was used for the measurement of fluorescence intensity. FRET measurements and analyses were performed as described previously (38, 39).

### Structural modeling analysis

Prediction of the integrin–talin1 complex structure and its energy minimization was performed using AlphaFold2 multimer (40). The interaction surface area of the integrin–talin1 complex in the predicted model was calculated using PISA (26).

### Octet analysis

Binding of the talin1 FERM domain to various synthetic peptides *in vitro* was measured using biolayer interferometry on an Octet RH96 system (Sartorius) (27). This label-free method monitors real-time changes in the interference pattern of light reflected from the biosensor surface, allowing direct quantification of binding kinetics. *Escherichia coli* BL21 (DE3) (Invitrogen) cells were transformed with pGEX-4T2 encoding GST or pGEX6P-1 encoding GST-fused talin1 FERM F2–F3 domain. The transformed cells were cultured at 30 °C until an absorbance reached 0.4 to 0.6 at 600 nm, at which point isopropyl β-d-1-thiogalactopyranoside (Wako) was added to a final concentration of 1 mM. After an additional 4 h of cultivation at 30 °C, the cells were harvested by centrifugation and lysed by sonication. GST and GST-fused talin1 FERM proteins were purified from the lysate using Glutathione Sepharose 4B (Cytiva). Purified proteins (10 μg/ml) were immobilized onto Octet GST Biosensors (Sartorius) as ligands. Synthetic integrin CT peptides (Fig. S4*B*) (GenScript) at concentrations of 4.9, 14.8, 44.4, and 133 μM, diluted in assay buffer (25 mM Tris–HCl, 150 mM NaCl, 0.05% Triton X-100, pH 7.5), were used as analytes. Binding responses were recorded, and kinetic parameters were calculated using Octet Analysis Studio. Nonspecific binding to GST alone was subtracted from GST-talin1 FERM binding signals before analysis.

## Data availability

All data are available in the main text or the supporting information.

## Supporting information

This article contains supporting information (15, 39).

## Conflict of interest

A patent application related to the findings reported in this article has been filed by the Kansai Medical University (Application number: 2025-137388). The patent covers the application of the identified WN linker sequence and its role in modulating integrin–talin interactions and adhesion. The authors confirm that the patent application does not restrict the availability of materials or data described in the article. The authors declare that they have no conflicts of interest with the contents of this article.

## Figures and Tables

**Fig S5:**