# Supplementary material for: The nonconserved integrin cytoplasmic region determines integrin subtype–specific characteristics by modulating talin1 binding kinetics
Source: J Biol Chem. 2025 Oct 7;301(11):110793. doi: 10.1016/j.jbc.2025.110793 (PMC12637224; doi:10.1016/j.jbc.2025.110793)
Supplement: Supporting information [file mmc1.docx]

Supporting Information for

The non-conserved integrin cytoplasmic region determines integrin subtype-specific characteristics by modulating talin1 binding kinetics

Naoyuki Kondo *et al.*

*Corresponding author. Email: [kondo.nao@kmu.ac.jp.](mailto:kondo.nao@kmu.ac.jp.)

**This file includes:**

Supplemental Figure legends (Figs. S1 to S5)

Movies S1 legend

References (1 to 2)

**Other Supplementary Materials for this manuscript include the following:**

Movies S1

**Supplemental Figure Legends**

Fig. S1.

**Establishment of *in vivo* single-molecule talin1 binding system and adhesion assay under shear flow conditions.** *A*, surface expression of α4 and αL integrins on CD3^+^ primary T cells. *B*, establishment of α4 (CD49d)-knockout (KO) BaF/3. Cells lacking β1, β2, β3, and β7 integrins established previously were used as the parental cells (Parental). α4-Deleted cells (α4-KO) were used as the new parental cells in this study to express intact and various chimeric integrins. *C*, adhesion assay on ICAM1-coated plastic dishes under shear flow conditions (0.5 dyn/cm^2^)(*1*) (αL/β2: *n* = 223; αLα4/β2β7: *n* = 122). Aliquots of 1×10^5^ cells were loaded. Adhesion events were divided into three categories, rolling (< 1 s), tether (1-3.3 s), and arrest (> 3.3 s), as described previously (*1*). *D*, dissociation rate constants of talin1 against β2 and β7 in both primary T cells and BAF/LFA1. The data of primary T cells from Fig. 1K are shown. *E*, expression of integrins and HT-talin1 in established BAF/LFA1 cells expressing αL-ΔGFFKR and either intact β2 or β2β7 chimera.

Fig. S2.

**Intramolecular FRET assay system to monitor tension applied to talin1.** *A*, schematic representation of the talin1 FRET tension sensor. A tandem fusion protein of YPet-FL peptide-mCherry was inserted at S447 in the linker between the FERM and ROD domains of talin1. The FL peptide is a component of a spring-like protein that extends in response to tension. FRET between YPet and mCherry occurs constitutively in the absence of tension. However, when tension is applied to the sensor, FRET decreases due to the separation of YPet and mCherry in response to the applied force. *B*, expression profile of integrins and talin1 tension sensor. Clone #27 was used for FRET and other analyses. *C*, acceptor bleaching experiment to verify the reliability of FRET changes in the cells (*2*). *D*, comparison of FRET signals between poly-l-lysine (PLL)-coated dishes, which do not induce ligand-dependent talin1 tension, and ICAM1-coated dishes in the presence of PMA (ICAM1+PMA), which induce talin1 tension generation via LFA1-dependent outside-in signaling. The entire area of cell adhesion was used for measurement of fluorescence intensity. Statistical analyses were performed using the unpaired nonparametric two-sided Student’s *t* test (*C*, *D*). ****p* < 0.001.

Fig. S3.

**Establishment of BAF/LFA1 cells expressing β integrin mutants and a system to measure ligand-induced conformational change.** The indicated integrins were introduced into Ba/F3 as established in Fig. 2B. *A*, FACS profile of BAF/LFA1 cells expressing β2-KQDS mutants. *B*, FACS profile of BAF/LFA1 cells expressing β7-NND mutants. *C*, expression levels of the KIM127 neoepitope in BAF/LFA1 cells treated with 100 ng/mL PMA or various concentrations of human ICAM1 (μg/mL). KIM127 signals were normalized to those of TS1/18 and are presented as fold change relative to unstimulated controls (normalized activation index). *n* = 4. *D*, species used in multiple sequence alignment (MSA) in Fig. 4A and phylogenetic analysis in Fig. 4B are listed. *E*, FACS profiles of BAF/LFA1 cells expressing wild-type (WT) β2 or β2-N749A mutant. Statistical analyses were performed using one-way ANOVA with Dunnett’s correction for multiple comparisons. ****p* < 0.001.

**Fig. S4.**

**Differences of integrin CTs and establishment of BAF/LFA1 cells expressing various integrin chimeras.** *A*, calculated interface area obtained using PISA analysis from five independently generated structural models of β2-, β7-, β1-, or β3-talin1 complexes. *B*, Octet biolayer interferometry analysis using GST-fused talin1 FERM domain and synthetic integrin-CT peptides (sequences shown at top). Non-specific binding of integrin-CT peptides to GST alone was subtracted from GST-talin1–integrin-CT binding signals. Binding parameters were determined using Octet Analysis Studio. *K*_d_: dissociation constant, *k*_on_: association rate constant, *k*_off_: dissociation rate constant. *C*, FACS profiles of BAF/LFA1 cells expressing αL-ΔGFFKR together with various chimeric β2 integrins. *D*, single-molecule binding analysis of talin1 to integrin-CT in β2-, or β2β1(β1)-expressing BAF/LFA1. Binding frequency (l*eft*, β2: *n* = 12; β1: *n* = 11) and binding duration (*right*, β2: *n* = 1046; β1: *n* = 835) of talin1 are shown. Frequencies of 1 to 10 s and > 10 s categories are shown in *yellow* and *red*, respectively. Statistical analyses were performed by one-way ANOVA with Tukey’s correction for multiple comparisons (*A*), the unpaired nonparametric two-sided Student’s *t* test (*D*) or Chi-square test (*D*). ****p* < 0.001.

Fig. S5

**Integrin expression, and PECAM1-dependent adhesion and talin1 binding.** *A*, RNA expression levels of α integrins in CD4^+^ or CD8^+^ T cells. Data from GENE SKYLINE (ImmuGen) were replotted. *B*, surface expression levels of αV and αL integrins in naïve T cells. *C*, adhesion assay for the natural integrin ligand PECAM1, which is presumably specific for αV/β3. Cell adhesion to PECAM1 (0.1 μg/mL: *n* = 42; 1 μg/mL: *n* = 41; 10 μg/mL: *n* = 55) was compared to ICAM1 (10 μg/mL: *n* = 48). A representative image of adhesion at 10 μg/mL PECAM1 is shown. *D*, single-molecule binding analysis of talin1 to the integrin-CT in αL/β2 or αV/β3 in primary T cells. (*left*) Binding frequency (ICAM1: *n* = 24; PECAM1: *n* = 35). (*right*) Binding duration (ICAM1: *n* = 1258; PECAM1: *n* = 290). Frequencies of 1 to 10 s and > 10 s categories are shown in *yellow* and *red*, respectively. *E*, adhesiveness of T-cell blasts onto PECAM1 (*n* = 45) and Th2-skewed cells onto ICAM1 (*n* = 52) and PECAM1 (*n* = 55). *F*, effects of β3 blocking antibody on adhesiveness onto PECAM1 (*n* = 59) in comparison with isotype control (*n* = 54). Statistical analyses were performed by one-way ANOVA with Tukey’s correction for multiple comparisons (*C, E*), the unpaired nonparametric two-sided Student’s *t* test (*D*, *F*) or Chi-square test (*D*). **p* < 0.05, ****p* < 0.001.

Movie S1.

Single-molecule imaging of HaloTag-fused talin1 in mouse primary T cells. Shown are movies captured under three conditions: ICAM1-coated surfaces with PMA stimulation (ICAM1), MAdCAM1-coated surfaces with PMA stimulation (MAdCAM1), and poly-l-lysine-coated surfaces (PLL). Time is indicated in the lower right corner in mm (minutes):ss (seconds) format. The arrow highlights a representative bright spot corresponding to talin1.

References

1. Y. Kamioka, Y. Ueda, N. Kondo, K. Tokuhiro, Y. Ikeda, W. Bergmeier, T. Kinashi, Distinct bidirectional regulation of LFA1 and alpha4beta7 by Rap1 and integrin adaptors in T cells under shear flow. *Cell Rep* **42**, 112580 (2023).

2. N. Kondo, Y. Mimori-Kiyosue, K. Tokuhiro, G. Pezzotti, T. Kinashi, The autophagy component LC3 regulates lymphocyte adhesion via LFA1 transport in response to outside-in signaling. *Nat Commun* **16**, 1343 (2025).
